# Exploitation of the Antibacterial Activity of *Micromeria graeca* L. Extracts From Northern Morocco

**DOI:** 10.1002/cbdv.202502410

**Published:** 2026-01-08

**Authors:** Houda Chtibi, Kaoutar Harboul, Taoufiq Benali, Aziz Bouymajane, Roberto Laganà Vinci, Francesco Cacciola, Luigi Mondello, Khalil Hammani

**Affiliations:** ^1^ Laboratory of Natural Resources and Environment Polydisciplinary Faculty of Taza Sidi Mohamed Ben Abdellah University of Fez Taza Morocco; ^2^ Laboratory of Ecotoxicology Bioresources and Coastal Geomorphology Polydisciplinary Faculty of Safi Cadi Ayyad University Safi Morocco; ^3^ Biology, Environment and Health Team Faculty of Sciences and Techniques of Errachidia Moulay Ismail University Meknes Morocco; ^4^ Messina Institute of Technology c/o Department of Chemical, Biological, Pharmaceutical and Environmental Sciences Former Veterinary School University of Messina Messina Italy; ^5^ Chromaleont S.r.l. c/o Department of Chemical, Biological, Pharmaceutical and Environmental Sciences Former Veterinary School University of Messina Messina Italy

**Keywords:** antimicrobial activities, chemical composition, *Micromeria graeca*, scanning electron microscopy, ultrasound‐assisted extraction

## Abstract

*Micromeria graeca* L. is an aromatic plant rich in bioactive molecules with potential antimicrobial properties. This study evaluated the antibacterial activity of extracts obtained using different solvents and extraction techniques. Among the tested samples, the methanolic ultrasound‐assisted extract (MGME‐UAE) exhibited the strongest bactericidal effect, selectively inhibiting *Proteus mirabilis* (inhibition zone 12.7 ± 0.6 mm; minimum inhibitory concentration [MIC] = 6.25 mg/mL). Liquid chromatography coupled with photodiode‐array and electrospray ionization–mass spectrometry (LC–PDA/ESI–MS) profiling revealed sagerinic acid (30.37 ± 0.70 mg/g), 5‐caffeoylquinic acid, and caffeic acid as major constituents. Mechanistic assays showed that MGME‐UAE increased membrane permeability, causing significant leakage of DNA/RNA and proteins, which was further confirmed by scanning electron microscopy (SEM) visualization of cell wall disruption. These findings highlight *M. graeca* as a promising source of natural antibacterial agents, particularly against *P. mirabilis*.

## Graphical abstract

Among the various extraction techniques investigated, the methanolic ultrasound‐assisted extract (MGME‐UAE) of *Micromeria graeca* L exhibited the strongest bactericidal effect, selectively inhibiting *Proteus mirabilis* (inhibition zone 12.7 ± 0.6 mm; MIC = 6.25 mg/mL). Sagerinic acid (30.37 ± 0.70 mg/g), 5‐caffeoylquinic acid, and caffeic acid, were determined by LC‐PDA/ESI‐MS as the major constituents. MGME‐UAE increased membrane permeability, causing significant leakage of DNA/RNA and proteins.

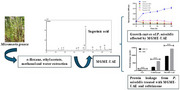

## 1 Introduction

Infectious diseases remain a major cause of morbidity and mortality worldwide, largely due to the emergence of resistant microbial strains resulting from the misuse and overuse of antimicrobial agents. This alarming trend has prompted an urgent search for novel, effective, and safer alternatives to conventional antimicrobials [1]. Medicinal and aromatic plants represent a rich source of bioactive compounds with potent antimicrobial properties and are increasingly being investigated as natural options for managing infectious diseases [2, 3, 4, 5, 6, 7, 8, 9]. The province of Taza, located in Northeastern Morocco, is characterized by remarkable plant biodiversity, owing to its varied topography and vast forest coverage, which accounts for 42.5% of its total area (468 000 ha) [10].

This study focuses on *Micromeria graeca*, a species belonging to the Lamiaceae family and part of the local flora of Taza, which is locally known as “Bakolt'nhal” [4]. The *Micromeria* genus comprises 130 perennial herb species [11], widely distributed across India, the Macaronesian–Mediterranean region, Southern Africa, and China [12, 13]. *Micromeria* species are used in traditional medicine for treating a range of ailments, including wounds, headaches, skin infections, asthma, colds, fever, and disorders of the respiratory and digestive systems [14]. They have also been reported to exhibit abortifacient, antiseptic, and anti‐rheumatic effects [12, 13]. The genus is further recognized for its antifungal, antimicrobial, anti‐inflammatory, antispasmodic, antiseptic, antitumor [11, 15, 16], and antioxidant properties [17, 18, 19]. *M. graeca* grows naturally in Taza, Northeast Morocco, where its leaf decoction is traditionally used as a substitute for mint. Our previous study demonstrated that its essential oil exhibits notable antioxidant potential and significant antimicrobial activity against pathogenic and phytopathogenic strains [5]. In addition, the aqueous extract has been reported to relax vascular smooth muscle through β‐adrenergic and cGMP pathways and exerts a potent antihypertensive effect [20]. It also displays anti‐hyperlipidemic and anti‐hyperglycemic activities in STZ‐induced diabetic rats, along with a powerful antioxidant capacity [21]. These biological activities are likely linked to its diverse polyphenol content [20]. Various extraction techniques, such as Soxhlet, maceration, and hydrodistillation, have been applied to recover polyphenolic compounds from plants. However, these traditional methods are often limited by high temperatures, long extraction times, and large solvent requirements, factors that can degrade thermolabile compounds and reduce extraction efficiency [22]. Consequently, advanced extraction methods such as ultrasound‐assisted extraction (UAE) and microwave‐assisted extraction have been developed. These innovative techniques offer several advantages, including lower operating temperatures, reduced solvent consumption, shorter extraction times, greater energy efficiency, and improved extraction yields [23, 24].

The present study aimed to evaluate the effects of different extraction techniques and solvents on the antibacterial activity of *M. graeca*. In particular, it sought to determine the polyphenolic profile of the most active extract and to elucidate its mechanism of antibacterial action. The research introduced a novel extraction strategy combining manual solvent fractionation with increasing polarity and ultrasonic treatment to enhance the recovery of active compounds. This optimized approach, applied here for the first time to *M. graeca* from Northern Morocco, provides valuable insight into the plant's bioactive potential and its role as a promising source of natural antibacterial agents.

## 2 Results and Discussion

### 2.1 Antibacterial Activity

The antibacterial potential of *M. graeca* extracts against common foodborne pathogens was assessed both qualitatively and quantitatively using the disk diffusion and triphenyl tetrazolium chloride (TTC) assays.

#### 2.1.1 Disk Diffusion Method

The antibacterial activity of different *M. graeca* extracts is summarized in Table 1. In vitro tests showed that the organic extracts from *M. graeca* were only effective against *Proteus mirabilis*. The antibacterial efficacy varied depending on both the solvent and extraction technique. Among the ultrasound‐assisted extracts, the methanolic extract (MGME‐UAE) exhibited the highest inhibitory activity, with an inhibition zone of 12.7 ± 0.6 mm. The hexane (MGHE‐UAE) and ethyl acetate (MGAE‐UAE) extracts showed comparable but slightly lower inhibition zones (10.7 ± 0.6 and 10.3 ± 0.6 mm, respectively). In contrast, maceration‐based extracts showed reduced activity, yielding inhibition zones of 6.7 ± 0.6 mm (MGHE‐Mac) and 8.0 ± 1.0 mm (MGME‐Mac). Interestingly, the ethyl acetate extract obtained by maceration (MGAE‐Mac) displayed higher inhibition (11.0 ± 1.0 mm) compared with its ultrasonic counterpart. The aqueous extracts (MGAqE), regardless of the extraction method, showed no detectable activity against any of the bacterial strains tested. Gentamicin, used as a positive control, produced a markedly larger inhibition zone (26 mm) against *P. mirabilis*. The observed differences in antibacterial activity are likely attributed to the distinct chemical compositions of the extracts, which vary depending on both the solvent polarity and extraction method. The outcomes from examining the antibacterial properties of various *M. graeca* extracts suggest that the MGME obtained via the UAE method displays a stronger and more extensive antimicrobial spectrum than the other extracts. This suggests that the extracts derived through the UAE technique possess greater efficacy than those obtained through maceration.

**TABLE 1:** Antibacterial activity of all extracts from *Micromeria graeca* against foodborne pathogen bacteria.

	Inhibition zone diameter (mm)				
Extracts 50 mg/mL	*Bacillus subtilis*	*Proteus mirabilis*	*Escherichia coli*	*Staphylococcus aureus*	*Pseudomonas aeruginosa*
MGAqE‐UAE	na	na	na	na	na
MGHE‐UAE	na	10.7 ± 0.6	na	na	na
MGAE‐UAE	na	10.3 ± 0.6	na	na	na
MGME‐UAE	na	12.7 ± 0.6	na	na	na
MGAqE Mac	na	na	na	na	na
MGHE Mac	na	6.7 ± 0.6	na	na	na
MGAE Mac	na	11.0 ± 1.0	na	na	na
MGME Mac	na	8.0 ± 1.0	na	na	na
**Gentamicin**	29 ± 0.0	26 ± 0.0	27 ± 0.0	27 ± 0.0	28 ± 0.0

*Note*: The diameter of the inhibition zones (mm) is given as a mean ± SD of triplicate experiments.

Abbreviations: Mac, maceration; MGAE, *M. graeca* ethyl acetate extract; MGAqE, *M. graeca* aqueous extracts; MGHE, *M. graeca* hexane extract; MGME, *M. graeca* methanolic extract; na, no activity; UAE, ultrasound.

Several studies have demonstrated that the extraction technique has a strong effect on both the extraction yield and the recovery of phenolic compounds. Previous studies, including our own [8] and those conducted by two different research groups [25, 26], have shown that UAE is a more effective method for polyphenol extraction. This enhanced efficiency is attributed to the propagation of ultrasonic waves, which involve cycles of compression and rarefaction. These waves generate cavitation bubbles that subsequently collapse within the solvent, disrupting plant cell walls and thereby facilitating mass transfer. As a result, the quantity of extracted material increases, ultimately improving the yield of target compounds [27, 28]. Moreover, prolonged ultrasonic exposure leads to a gradual increase in the temperature of the water bath due to sound radiation [29]. This temperature rise aids in the extraction process by decreasing solvent viscosity and increasing diffusion and solubility coefficients [30]. Moreover, the selection of solvents is significant in the study of phytochemical compounds. Solvent selection is also a critical factor in phytochemical extraction. Previous studies have employed various solvents, including *n*‐hexane, chloroform, water, methanol, ethyl acetate, and ethanol [8, 14, 31, 32, 33]. These studies consistently demonstrate that methanol is one of the most effective solvents for extracting phenolic compounds. Ethanolic extracts typically contain higher concentrations of phenolics and flavonoids compared with other solvent extracts. Consequently, the notable antibacterial activity observed in methanolic extracts may be attributed to their elevated phenolic content. These findings differ from those of El Kamari et al. [34], who reported significant antifungal activity of the aqueous *M. graeca* extract against *Candida albicans* and *Aspergillus niger* (inhibition zone = 16 mm). Similarly, El Khoury et al. [35] observed that *M. graeca* aqueous extracts strongly inhibited aflatoxin B1 biosynthesis by *Aspergillus flavus*. In contrast, our results indicate that aqueous extracts lack antibacterial efficacy against the bacterial strains tested. Moreover, Benali et al. [5] demonstrated potent antibacterial activity of *M. graeca* essential oil, which exhibited broad‐spectrum effects but lacked the selectivity observed in the methanolic extract. Such discrepancies are likely due to differences in chemical composition, particularly the predominance of volatile, lipophilic compounds in essential oils versus polar constituents in solvent extracts.

#### 2.1.2 Minimum Inhibitory Concentration (MIC) and Minimum Bactericidal Concentration (MBC)

MIC values ranged from 1.56 mg/mL, observed for both the MGAE‐US extract and the MGHE maceration extract, to 12.5 mg/mL for the MGME maceration extract. Although the MIC values of the MGME extracts obtained by both extraction techniques were relatively high, they exhibited a bactericidal effect, as indicated by an MBC/MIC ratio equal to 4. In contrast, the remaining extracts showed a bacteriostatic effect, characterized by MBC/MIC ratios greater than 4 (Table 2). Our results are in agreement with previous findings by Benali et al. [5], who reported MIC values for *M. graeca* essential oil ranging from 1.56 to 12.5 mg/mL against the same bacterial strains tested in the present study. Specifically, the MIC of the essential oil against *P. mirabilis* was found to be 3.125 mg/mL. Similar trends have been documented for *Micromeria cristata* essential oils from Turkey, which exhibited MIC values between 31 and 250 µg/mL against a variety of pathogens [36]. In contrast, polar extracts of *Micromeria* species generally exhibit weak antibacterial activity, as shown by Ali‐Shtayeh et al. [37], who observed minimal activity of aqueous *Micromeria nervosa* extracts. Likewise, Brahmi et al. [11] reported that ethanolic *M. graeca* extracts had MIC values exceeding 2000 µg/mL.

**TABLE 2:** Minimum inhibitory concentration (MIC) and minimum bactericidal concentration (MBC) values (mg/mL) of extracts of *Micromeria graeca*.

	MIC (mg/mL)	CMB (mg/mL)
Extracts/Strain	*Proteus mirabilis*	*Proteus mirabilis*
MGHE‐UAE	3.125	>50
MGAE‐UAE	1.56	>50
MGME‐UAE	6.25	25
MGHE Mac	1.56	25
MGAE Mac	6.25	50
MGME Mac	12.5	50

Abbreviations: Mac, maceration; MGAE, *M. graeca* ethyl acetate extract; MGAqE, *M. graeca* aqueous extract; MGHE, *M. graeca* hexane extract; MGME, *M. graeca* methanolic extract; UAE, ultrasound.

The stronger antibacterial activity of essential oils compared with polar extracts is likely attributable to the lipophilic nature of their active constituents, which readily diffuse through bacterial membranes. The molecular structure and degree of hydrophobicity of these compounds play crucial roles in enhancing membrane permeability and disrupting membrane integrity, ultimately leading to bacterial cell death [11].

#### 2.1.3 Chemical Components of Extracts Using Liquid Chromatography Coupled With Photodiode‐Array and Electrospray Ionization–Mass Spectrometry (LC–PDA/ESI–MS)

**FIGURE 1:**
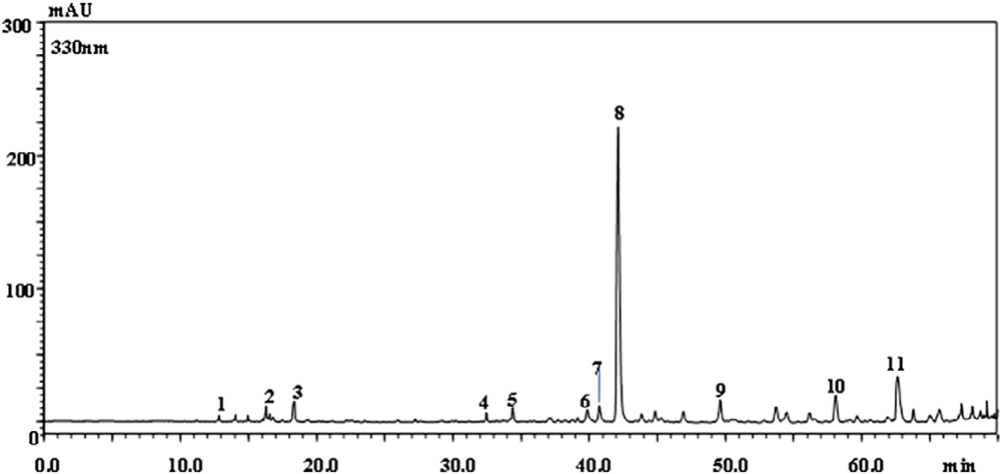
LC chromatogram (*λ* = 330 nm) of the MGME‐UAE.

Given that the methanolic extract obtained by ultrasound‐assisted extraction (MGME‐UAE) exhibited the strongest antibacterial activity (Table 1), it was subjected to detailed chemical characterization by LC–PDA/ESI–MS [38]. The chromatogram (*λ* = 330 nm) and the identified polyphenolic constituents are shown in Figure 1 and Table 3, respectively. Figure 1 and Table 3 report the LC chromatogram (*λ* = 330 nm) and the identified polyphenolic compounds, respectively. In total, up to 11 polyphenolic components were identified and quantified (mg/g). The compound with the highest concentration was the sagerinic acid (30.37 ± 0.70 mg/g), followed by 5‐caffeoylquinic acid (3.52 ± 0.11 mg/g), caffeic acid (2.20 ± 0.10 mg/g), luteolin glucuronide (0.46 ± 0.01 mg/g), and apigenin glucuronide (0.38 ± 0.03). Salvianolic acid B/E/L, salvianolic acid J, and lithospermic acid were detected but not quantified. Sagerinic, caffeic, and 5‐caffeoylquinic acids are known for a wide range of pharmacological effects, particularly antimicrobial activity. The propene side chain of caffeic acid enhances its hydrophobicity, enhancing its ability to penetrate bacterial membranes and contributing to bactericidal effects against *Staphylococcus aureus* and *Escherichia coli*, comparable to those of ampicillin [39]. Similarly, several phenolic acids, including caffeic acid isolated from wild Polish mushrooms, demonstrated moderate antibacterial activity against both Gram‐positive and Gram‐negative strains [40]. Due to their weak acidic nature, these compounds can diffuse through bacterial membranes, causing cytoplasmic acidification and eventual cell death [41]. Caffeic acid has also been widely associated with antimicrobial effects. For instance, extracts from *Potentilla* species rich in caffeic acid exhibited strong antibacterial effects against a broad spectrum of bacteria [42]. Chlorogenic acid, an ester of quinic and caffeic acids, also exerts bactericidal effects by inducing physiological alterations in microbial cell membranes [43]. Several studies from Algeria reported caffeic acid and apigenin glucuronide among the major constituents of *M. graeca* extracts, together with chlorogenic, gallic, rosmarinic, and diosmin acids [11, 44].

**TABLE 3:** Phenolic compounds in the MGME‐UAE identified through liquid chromatography coupled with photodiode‐array and electrospray ionization–mass spectrometry (LC–PDA/ESI–MS) analysis.

Peak no.	Compound	*t*_R_ (min)	UV max (nm)	[M − H]^−^	Methanolic extract	Ref.
1	3‐Caffeoylquinic acid	12.97	323	353, 179	0.86 ± 0.02	[47]
2	Caffeic acid	16.29	322	179	2.20 ± 0.10	—
3	5‐Caffeoylquinic acid	18.39	325	353, 179	3.52 ± 0.11	[47]
4	Salvianolic acid J	32.53	255, 283sh, 342	537	X	[48]
5	Luteolin glucuronide	34.40	257, 341	461, 285	0.46 ± 0.01	[49]
6	Apigenin glucuronide	39.99	266, 333	445, 269	0.38 ± 0.03	[50]
7	Isovitexin sinapoylhexoside	35.66	261, 337	799	—	—
8	Sagerinic acid	42.29	327	719, 359	30.37 ± 0.70	[48]
9	Salvianolic acid B/E/L	49.76	286, 329	717	X	—
10	Lithospermic acid A	58.17	285, 329	537	X	—
11	Salvianolic acid B/E/L isomer	62.63	285, 329	717	X	—

*Note*: Quantitative data are reported as mg/g. X: detected but not quantified; sh: wavelength shoulder. Quantitative data are reported as milligrams per gram (mg/g) of dried extract ± SD (*n* = 3).

Other *Micromeria* species have also been characterized by diverse polyphenolic profiles. Ethanolic extracts of *Micromeria croatica*, *Micromeria juliana*, and *Micromeria thymifolia* contain flavonoids such as apigenin and luteolin and phenolic acids, including rosmarinic and chlorogenic acids [19]. Likewise, *M. nervosa* extracts contained rosmarinic, vanillic, ferulic, syringic, and caffeic acids, along with flavones such as luteolin and apigenin [12], whereas *Micromeria parviflora* was reported to contain rosmarinic, rutin, chlorogenic, and caffeic acids [44]. Methanolic and aqueous extracts of *Micromeria myrtifolia* also exhibited significant quantities of syringic, rosmarinic, and protocatechuic acids [13]. Flavones, particularly luteolin and apigenin, are recognized for their ability to inhibit bacterial efflux pumps, mechanisms commonly associated with antimicrobial resistance in Gram‐positive bacteria [45]. These compounds have demonstrated moderate antibacterial activity (MIC ≈ 500 mg/L) against clinical isolates of both Gram‐positive and Gram‐negative bacteria [46].

Collectively, these findings confirm that *M. graeca* methanolic extracts are rich in phenolic acids and flavones with well‐established antimicrobial potential. The predominance of sagerinic, caffeic, and chlorogenic acid derivatives likely contributes to the pronounced antibacterial efficacy observed for MGME‐UAE.

### 2.2 Mechanisms of Antibacterial Action

#### 2.2.1 Antibacterial Kinetics Assay

**FIGURE 2:**
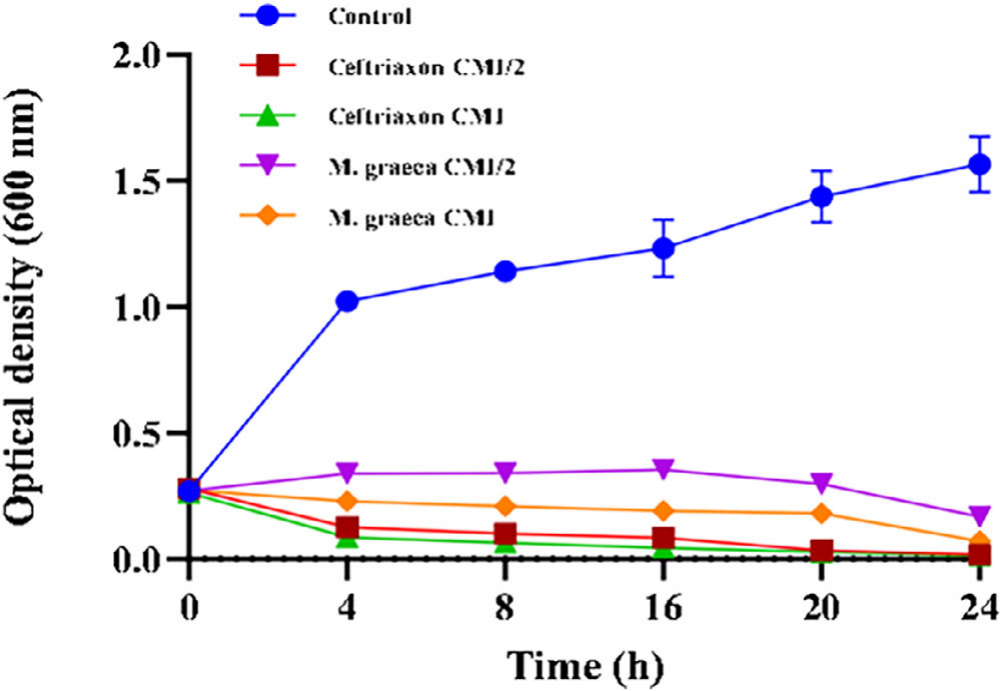
The growth curves of *Proteus mirabilis* affected by the MGME extract obtained by UAE of *Micromeria graeca*.

The bactericidal kinetics of the methanolic ultrasound‐assisted extract (MGME‐UAE) against *P. mirabilis* were evaluated to determine its time‐dependent antibacterial activity (Figure 2). In the absence of treatment, *P. mirabilis* exhibited normal growth, with optical density (OD_600_) increasing from 0.3 to 1.0 within 4 h. In contrast, significant growth inhibition was observed when cultures were treated with MGME‐UAE or ceftriaxone at concentrations corresponding to the MIC and half the MIC (MIC/2).

At MIC/2, the extract delayed bacterial growth up to the 16th hour, followed by a sharp decline in OD, indicating progressive cell death. Treatment at the MIC concentration resulted in immediate inhibition of bacterial proliferation. For comparison, ceftriaxone achieved complete bacterial eradication within 4 h at both MIC and MIC/2, as reflected by a reduction in OD from 0.3 to 0.085. MGME‐UAE produced a similar bactericidal effect within 24 h, demonstrating concentration‐dependent inhibition.

Time‐kill assays confirmed that MGME‐UAE exerted both concentration‐ and time‐dependent antibacterial effects. Such effects are consistent with mechanisms involving the interaction of bioactive compounds with bacterial cell membranes, leading to structural damage, leakage of intracellular contents, and eventual cell death [51, 52].

#### 2.2.2 Cell Membrane Integrity

##### 2.2.2.1 Leakage of Nucleic Acid

**FIGURE 3:**
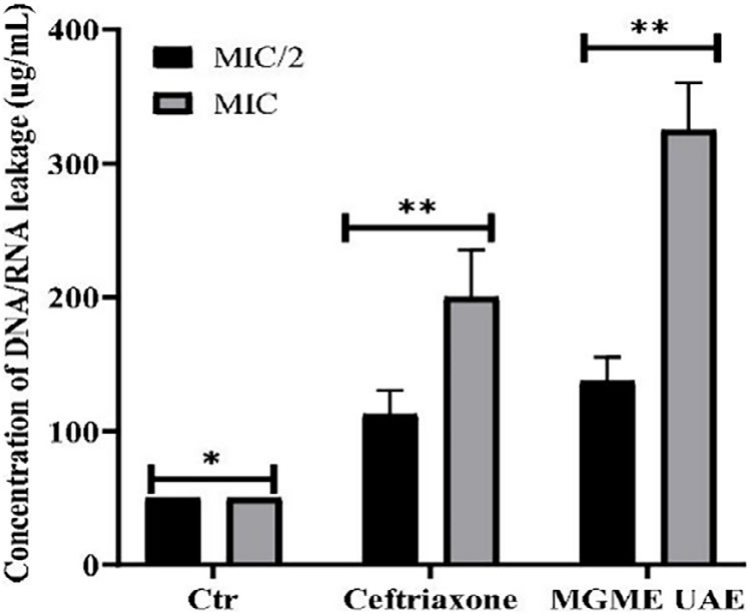
DNA/RNA leakage from *Proteus mirabilis* after being treated with MGME‐UAE and ceftriaxone (positive control) at MIC/2 and MCI concentrations. Untreated cells (Ctr): negative control. Data are presented as mean (±SD) of three replicates (compared with the control, *p < 0.05; **p < 0.01). MIC, minimum inhibitory concentration.

The impact of MGME‐UAE on the cell membrane integrity was evaluated by the measurement of the quantity of DNA/RNA released [53]. The release of the nucleic acid from bacteria, *P. mirabilis*, treated with MGME‐UAE was determined by the measurement of the absorbance of the supernatant of the bacterial culture at 260 nm. According to the results shown in Figure 3, the leakage of DNA/RNA from bacteria exposed to MGME‐UAE and ceftriaxone increased significantly in a dose‐dependent manner compared to untreated cells (50.00 µg/mL). MGME‐UAE induced an important release of nucleic acid from *P. mirabilis*. The concentrations of DNA released from *P. mirabilis* treated with MGME‐UAE and ceftriaxone at MIC/2 concentration were 137.5 ± 17.67 and 112.5 ± 17.67 µg/mL, respectively. At a high concentration (MIC concentration), the genetic material release is more interesting: 325 ± 35.35 µg/mL (MGME‐UAE) and 200 ± 35.35 µg/mL (ceftriaxone). This indicates that the MGME‐UAE results in greater leakage of DNA/RNA after the disruption of bacterial cell membranes. Interestingly, the MIC of MGME‐UAE exhibited the maximum nucleotide leakage with 325 ± 35.35 µg/mL, higher than ceftriaxone (positive control); this indicates that the increasing permeability of the bacterial plasma membrane had instigated the leakage of intracellular contents from the bacterial cells. These alterations ultimately directed to the cell death.

##### 2.2.2.2 Leakage of Intracellular Proteins

**FIGURE 4:**
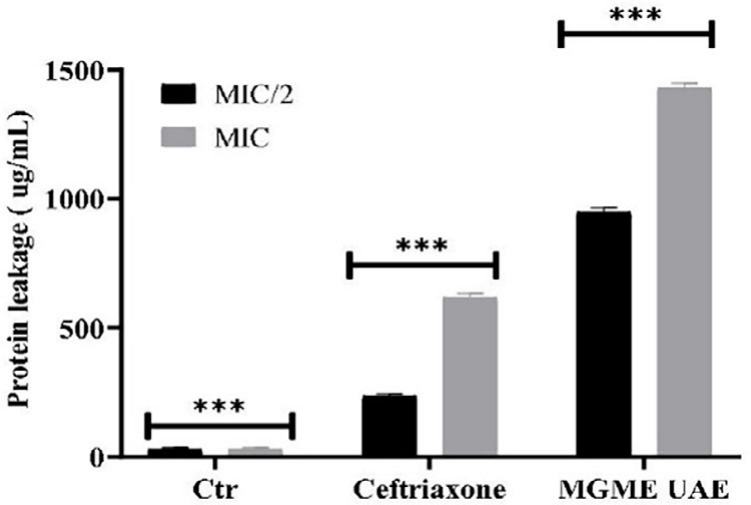
Protein leakage from *Proteus mirabilis* treated with MGME‐UAE and ceftriaxone (positive control) with CMI and CMI/2 concentrations, compared to the untreated cells (negative control). Each point represents the mean values of three experiments (*n* = 3). MIC, minimum inhibitory concentration. ***p < 0.001.

Protein leakage assays further confirmed the membrane‐disruptive action of MGME‐UAE. As shown in Figure 4, extracellular protein concentrations increased in a dose‐dependent manner. Treatment with MGME‐UAE resulted in protein leakage of 949.3 ± 15.0 and 1430 ± 18.6 µg/mL at MIC/2 and MIC, respectively. In contrast, ceftriaxone induced lower leakage levels of 236.3 ± 9.0 µg/mL (MIC/2) and 617 ± 15 µg/mL (MIC). The untreated control showed minimal leakage (29.7 ± 6.8 µg/mL).

These results demonstrate that MGME‐UAE significantly compromises the bacterial membrane, leading to extensive efflux of cytoplasmic proteins. The higher leakage induced by the extract compared to ceftriaxone suggests that MGME‐UAE causes greater membrane destabilization, supporting its potential as a natural antibacterial agent capable of inducing cell lysis.

The bacterial cytoplasmic membrane acts as a selectively permeable barrier essential for maintaining ion homeostasis, energy status, and metabolic balance [54, 55]. Disruption of this barrier alters ion gradients, interferes with energy‐dependent processes, and leads to leakage of macromolecules such as DNA and proteins, ultimately causing cell death [56, 57].

In the present study, MGME‐UAE caused significant leakage of both nucleic acids and proteins, confirming its ability to disrupt the cytoplasmic membrane of *P. mirabilis*. Similar effects have been reported for plant‐derived polyphenols, which can alter membrane potential, increase permeability, and induce pore formation [58, 59, 60, 61, 62, 63]. These compounds may integrate into the lipid bilayer, modify its fluidity, or interact with membrane proteins through hydrogen bonding or hydrophobic interactions, leading to loss of structural integrity [58, 59].

The hydroxylation pattern of flavonoids plays a critical role in these interactions: Highly hydroxylated, lipophilic flavonoids are particularly disruptive to bacterial membranes [64, 65, 66]. Phytochemical analysis of MGME‐UAE revealed the presence of such phenolic compounds, including luteolin, caffeic acid, and apigenin glucuronide, each known to interfere with bacterial membrane structure and function.

Luteolin has been shown to cause morphological damage, membrane disruption, and inhibition of Topoisomerase I/II in *Listeria monocytogenes* and *S. aureus*, leading to decreased ATP synthesis and impaired energy metabolism [59, 67, 68, 69, 70, 71, 72, 73]. Similarly, caffeic acid alters the permeability of both the plasma and outer membranes of *Pseudomonas aeruginosa*, resulting in leakage of DNA and potassium ions essential for cell viability [74, 75, 76, 77]. Apigenin and its derivatives are known to target key enzymes involved in nucleic acid and cell wall synthesis, such as d‐alanine‐d‐alanine ligase and components of the Type II fatty acid synthesis pathway [78, 79, 80].

Thus, the strong bactericidal activity of MGME‐UAE likely arises from synergistic interactions among these phytochemicals, amplifying their effects on membrane integrity and bacterial metabolism [80, 81].

#### 2.2.3 Morphological Modifications of MGME‐UAE Against *Proteus mirabilis* Using Scanning Electron Microscopy (SEM)

**FIGURE 5:**
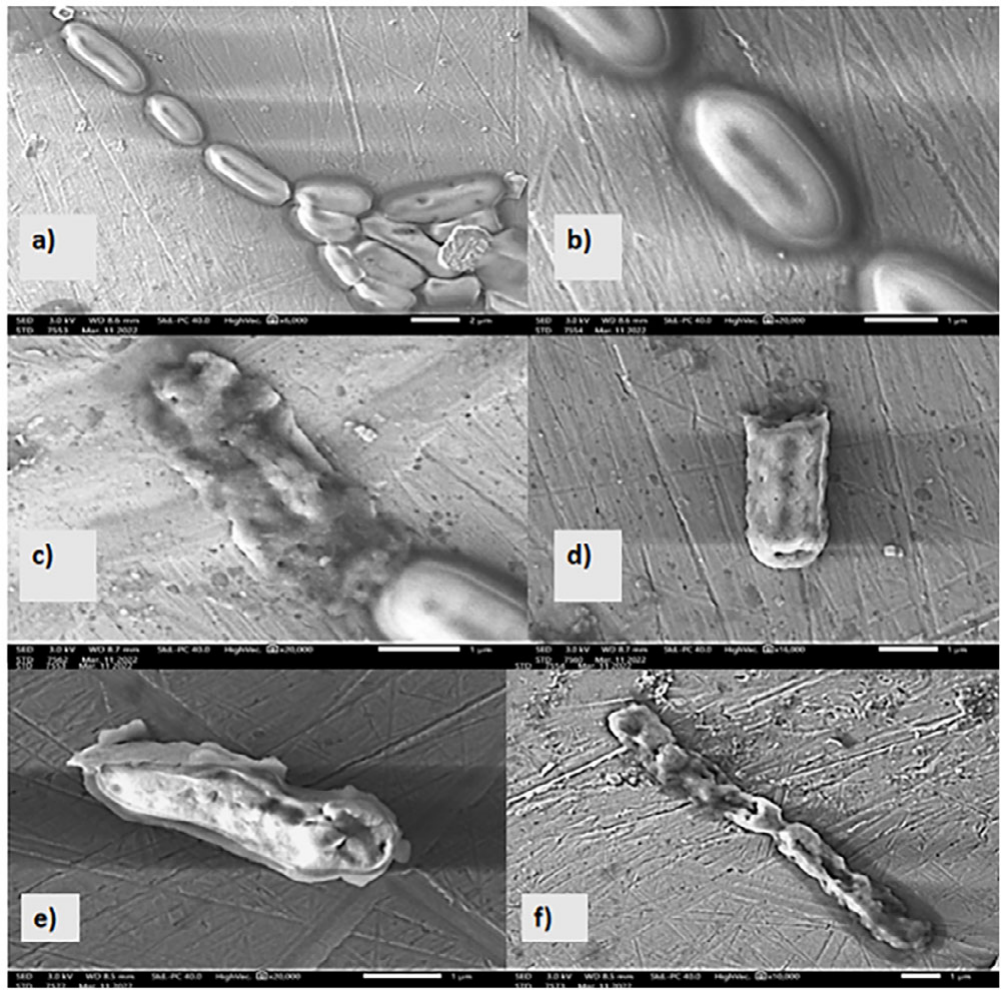
SEM micrographs of *Proteus mirabilis* cells treated with *Micromeria graeca* methanolic extract (origin, Morocco). Panels (a) and (b): Images of untreated cells, with magnifications gradually increasing (×6000, ×20 000); Panels (c) and (d): cells exposed to the methanolic extract obtained via UAE at MIC value, with magnification of ×16 000, ×20 000; Panels (e) and (f): cells treated with the methanolic extract obtained through maceration at MIC value, with magnification of ×10 000, ×20 000.

To understand the effect of *M. graeca* extract on the sensitive bacteria, *P. mirabilis*, the scanning electron microscope (SEM) was used to visualize the morphological alterations of *P. mirabilis* cells that were exposed to methanolic extracts derived from the two extraction techniques (Figure 5). Untreated control cells exhibited smooth, intact, rod‐shaped surfaces with no visible deformations (Figure 5a,b). In contrast, cells treated with MGME‐UAE at the MIC concentration displayed severe morphological alterations, including irregular shapes, surface roughness, and distinct perforations in the cell wall (Figure 5c–f).

Analysis of the SEM images shows that both tested extracts produced similar morphological effects on *P. mirabilis* cells. However, the extract obtained by maceration requires twice the concentration of the extract obtained by UAE to achieve the same effect. This can maybe be explained by the presence of a greater quantity of antibacterial molecules in the extract obtained by MGME‐UAE than in the extract obtained by maceration, which is responsible for this effect. Therefore, the UAE technique is more effective than the maceration technique at extracting bioactive molecules. These results agree with our previous study [8], in which we found that the methanolic extract obtained by sonication was the only one that presented a bactericidal effect against *P. mirabilis*. These findings align with those reported by Latha et al. [82] who demonstrated that *Vernonia cinerea* hydro‐methanolic extract could effectively destroy bacterial cells and inhibit the growth of *P. aeruginosa*. In addition, the study conducted by Selvi et al. [83] utilized SEM to examine the effects of the methanolic leaf extract of *Bixa orellana* on *P. aeruginosa* cells. The results showed that the cells underwent disintegration and aggregation after treatment, indicating cell lysis and death. Moreover, the study by Kaya et al. [84] showed that the *P. aeruginosa* cells treated with the methanolic extracts of *Ocimum basilicum* appeared to shrink and there was a degradation of the cell walls. *P. mirabilis* is a Gram‐negative bacterium characterized by an outer membrane composed of lipopolysaccharides. This membrane serves as a barrier to numerous hydrophilic compounds, including antibiotics. The impermeability of this membrane to bactericidal agents could be a possible cause of antibiotic resistance. However, phytochemical molecules can work together in synergy; for example, one compound can modulate cell membrane permeability, making it easier for another compound to disrupt the bacterium's metabolic processes.

**FIGURE 6:**
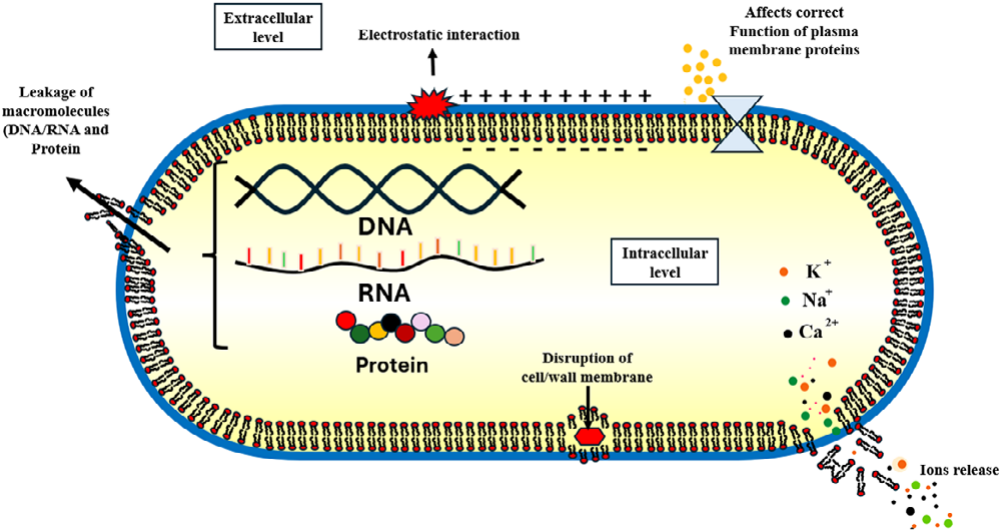
Proposed antibacterial mechanisms of MGME‐UAE against *Proteus mirabilis*.

The obtained results suggest that the observed damage to the cytoplasmic membrane is irreversible, leading to the leakage of cellular constituents (DNA/RNA and proteins) and ultimately resulting in bacterial lysis, as illustrated in Figure 6.

## 3 Conclusions

This study demonstrated that both the extraction method and solvent type significantly influence the antibacterial activity of *M. graeca* extracts collected from Northern Morocco. Among all tested samples, the methanolic extract obtained by ultrasound‐assisted extraction (MGME‐UAE) exhibited the strongest antibacterial potential, evidenced by the largest inhibition zone and the lowest MIC value against *P. mirabilis*. LC–PDA/ESI–MS analysis revealed that this extract is particularly rich in phenolic acids, including sagerinic, caffeic, and 5‐caffeoylquinic acids, as well as flavone derivatives such as luteolin and apigenin glucuronides. These metabolites are known to exert broad‐spectrum antimicrobial effects by disrupting cell membrane integrity, inhibiting nucleic acid synthesis, and interfering with bacterial energy metabolism.

Mechanistic investigations confirmed that MGME‐UAE induces irreversible damage to *P. mirabilis* cell membranes, leading to substantial leakage of DNA/RNA and proteins, along with pronounced morphological alterations observed by SEM. These findings suggest that the extract's bactericidal activity arises from synergistic interactions among multiple phytoconstituents rather than from a single dominant compound.

Overall, this work highlights *M. graeca* as a promising natural source of antibacterial agents. UAE proved particularly effective for producing bioactive‐rich extracts while preserving compound stability. The results provide a solid scientific basis for the potential application of *M. graeca* extracts in developing novel plant‐derived antimicrobial formulations and underscore the need for further studies on their mechanisms of action and therapeutic potential.

## 4 Experimental Section

### 4.1 Plant Material

Plant was collected from Taza, in Northern Morocco (004°52.607′ N, 004°01.190′ W and 34°09.825′ N, 004°09.850′ W). The identification of plants was achieved by Pr. Ennabili Abdeslam and Dr. Khabbach Abdelmajid in the Natural Resources and Environment Laboratory of the Polydisciplinary Faculty of Taza, Sidi Mohamed Ben Abdellah University of Fez, and a voucher specimen (FPT‐LRNE‐72). Subsequently, the plant was dried at room temperature in the gloom until it reached a constant weight, then ground into a powder using an electric mill, ensuring it passed through a 0.5 µm sieve. *M. graeca* was identified as described in our previous study [4].

### 4.2 Preparation of the Extracts

Our study used a manual fractionation extraction process. First, 20 g of powder from the plant's aerial parts, comprising leaves, stems, and flowers, was extracted with 100 mL of hexane. The remaining residue went through multiple re‐extractions with solvents of increasing polarity. This step‐by‐step extraction method helped us categorize compounds based on their polarity.

The plant material was extracted using two methods, namely, maceration and sonication. Maceration involved occasional stirring for 72 h at room temperature, whereas sonication was performed in an ultrasonic bath for 60 min at 25°C. After extraction, the crude extract was filtered and concentrated using a rotary vacuum evaporator to obtain the organic extract. The aqueous extract was freeze‐dried and stored in darkness at a temperature of +4°C until further testing.

### 4.3 Antimicrobial Activity Evaluation

#### 4.3.1 Bacterial Strains and Growth Conditions

The antimicrobial activity of the extracts was tested against a selection of both Gram‐positive bacteria (*S. aureus* CECT 976 and *Bacillus subtilis* DSM 6633) and Gram‐negative bacteria (*P. aeruginosa* CECT 118, *E. coli* K12, and *P. mirabilis*), which are commonly associated with foodborne infections. These bacterial strains were obtained from the Laboratory of Biology and Health at the Faculty of Sciences in Tetouan. Bacterial cultures were prepared by cultivating the strains on Mueller–Hinton agar (MHA) for 24 h at 37°C. The inoculum test concentration for the strains was standardized at 106 CFU/mL.

#### 4.3.2 Disk Diffusion Method

The disk diffusion method was utilized to evaluate the antibacterial activity, based on a previous protocol [3]. In this method, Whatman paper discs with a diameter of 6 mm were placed onto MHA, previously inoculated with bacteria. Each disc was then loaded with extract solution at a concentration of 50 mg/mL. Additionally, a positive control of gentamicin (15 µg) and a negative control of 10% DMSO were also tested. After a 2‐h incubation period at room temperature to allow for extract diffusion, the plates were further incubated for 24 h at 37°C, and the diameter of the inhibition zones was measured to assess antibacterial activity.

#### 4.3.3 Determination of MIC and MBC

To determine the MICs, the active extracts were subjected to testing using the microdilution technique. This method followed the protocol outlined by Benali et al. [3]. A sterile 96‐well microplate was used for the assay. To initiate the assay, 100 µL of Mueller–Hinton Broth (MHB) was added to each well, except the first well, which contains 200 µL of MHB and extract at 50 mg/mL; then serial dilution was performed by transferring 100 µL of the solution from the first well to the subsequent wells, creating a decreasing concentration gradient ranging from 50 to 0.019 mg/mL. Finally, 10 µL of a bacterial suspension containing 10^6^ CFU/mL was added to each well. The tenth well served as a sterile control, and the 11th served as a positive growth control (bacteria only), whereas the last well was used as a negative control (DMSO, 10%). After 24 h of incubation at 37°C, 20 µL of 5 mg/mL of TTC, which possesses the property of remaining colorless in areas where bacterial growth is absent, was added to each well. The plates were then re‐incubated at 37°C for an additional 2–4 h. The MIC was determined to be the lowest extract concentration that inhibited visible bacterial growth. To determine the MBC, 20 µL of broth from the uncolored wells was plated onto MHA plates and incubated for 24 h at 37°C.

### 4.4 Analysis of the Polyphenolic Compounds by LC–PDA–ESI/MS

#### 4.4.1 Sample Preparation

Before being subjected to HPLC‐PDA‐ESI/MS analysis, dried extract of *M. graeca* was re‐dissolved in a methanolic solvent and filtered (0.45 µm nylon membrane).

#### 4.4.2 HPLC–MS Analysis Conditions

Chromatographic analyses were performed using a Shimadzu HPLC system, equipped with a CBM‐20A controller, LC‐30AD pumps, DGU20A5 R degasser, CTO‐20AC oven, SIL‐30AC autosampler, SPD‐M20A detector, and LCMS‐2020 mass spectrometer [85]. Chromatographic separations were performed using an Ascentis Express C18 column (150 × 2.1 mm^2^, 2.7 µm) from Merck Life Science, Merck KGaA, Darmstadt, Germany. The mobile phase consisted of 0.1% formic acid in water (A) and acetonitrile (B). The gradient of elution was employed, starting from 0 min—0%, 10 min—10% B, 10 min—15% B, 15 min—13% B, 25 min—16% B, 30 min—20% B, 55 min—26% B, 65 min—32% B, and 70 min—100% B, at a flow rate of 0.5 mL/min. A 2 µL sample was injected, and PDA detection was performed from 200 to 400 nm, with chromatograms extracted at 330 nm. MS spectra were acquired from *m*/*z* 100–1200, and data acquisition was performed using Shimadzu LabSolution software.

#### 4.4.3 Chromatographic Method Validation

The chromatographic method was validated by assessing linearity, repeatability, extraction recoveries at two enrichment levels, limits of detection (LOD), and limits of quantification (LOQ).

Calibration curves for 4‐caffeoylquinic acid, apigenin, kaempferol‐3‐glucoside, and rosmarinic acid were generated by injecting 5 µL of standard solutions at five different concentrations (0.1–100 mg/L) in six replicates. LOD and LOQ were determined by analyzing three injections of a standard mixture at three concentration levels (0.01, 0.05, and 0.08 mg/L) and calculating the signal‐to‐noise ratios of 3:1 and 10:1, respectively.

To evaluate the recovery of phenolics, an *Inula viscosa* sample, previously confirmed to be devoid of the target phenolic compounds [86], was used as an external matrix. Known amounts of the four standards were added to a 1 g sample of *I. viscosa*, and the extraction procedure was carried out following the same protocol used for *M. graeca*.

Intraday repeatability of retention times (*t*_R_) and relative areas was evaluated by analyzing a standard mixture of the five phenols five times on the same day. For interday repeatability determination, retention times and relative areas were measured at two different concentration levels of the five phenols over three different days.

### 4.5 Mechanisms of Antibacterial Action

#### 4.5.1 Antibacterial Kinetics Assay

The bactericidal efficacy of the most potent extract was investigated using a growth curve assay. Susceptible bacterial strains were exposed to the extract at the MIC and half the MIC (MIC/2). Ceftriaxone, a beta‐lactam antibiotic, was employed as a positive control at MIC and 0.5 × MIC. Bacterial growth was monitored spectrophotometrically at 600 nm at 0, 4, 8, 16, 20, and 24 h at 37°C. All experiments were conducted in triplicate.

#### 4.5.2 Cell Membrane Integrity

##### 4.5.2.1 Leakage of Nucleic Acid

The integrity of the cell membrane was evaluated by the quantification of the DNA released from *P. mirabilis* cells, with minor modification [54]. The overnight cells of *P. mirabilis* were centrifuged at 4000 rpm for 15 min; pellets were rinsed and resuspended in PBS (10 mM, pH 7.4) and adjusted to OD_600_ to 0.7, then treated for 24 h at 37°C with MGME‐UAE at different concentrations of CMI and CMI/2; the positive control was ceftriaxone with the same concentration as MGME‐UAE, and the negative control was the cells without treatment. After treatment, the tubes were centrifuged at 6000 rpm for 15 min at 4°C, and the supernatant was collected for the measurement of the OD at 260 nm.

##### 4.5.2.2 Leakage of Intracellular Proteins

The protein leakage was utilized for the evaluation of the cell membrane integrity; the experiments were planned on the basis of the study of Bouyahya et al. [54] with slight modifications. The quantity of proteins that are released into the extracellular medium was performed using the Lowry assay. In brief, the bacterial cells of *P. mirabilis* were taken and centrifuged for 15 min at 4000 rpm; the pellets were rinsed with a phosphate buffer of 0.1 M, which had a pH of 7. Furthermore, the pellet was resuspended in the PBS buffer and was adjusted OD_600_ to 0.7, then treated, for 24 h at 37°C, with MGME‐UAE at different concentrations of CMI and CMI/2; the positive control was ceftriaxone at the CMI and CMI/2 concentrations, and the negative control was the cells without treatment. After treatment, the tubes were centrifuged at 6000 rpm for 15 min, and the supernatant was collected for the Lowery analysis.

### 4.6 Scanning Electron Microscopy

To elucidate the mechanism of action, the morphology of bacteria treated with the bactericidal extract was examined using SEM. This approach was based on previous literature reports [87, 88, 89]. Only bacterial strains susceptible to the extracts were selected. To perform the SEM analysis, bacterial cells were adjusted to McFarland 1 turbidity and treated with the most effective extract at its MIC. After the incubation period, the cells were harvested by centrifugation for 15 min at 7000 rpm in 4°C. The bacterial pellet was then washed twice with sterile potassium nitrate solution and resuspended.

A 20 µL droplet of each suspension was spread on a microscope slide, air‐dried, and coated with gold for SEM analysis. Untreated bacteria served as a negative control. The dried samples were then deposited on conductive and adhesive stainless‐steel slides, coated with a layer of gold under vacuum, and examined using a scanning electron microscope (ISM‐IT500HR) to visualize the cell morphology.

### 4.7 Statistical Analysis

The results were statistically analyzed, which involved repeating the tests three times. The software used for statistical analysis was XLSTAT Version 2016.02.28451. The mean and standard error were calculated to summarize the data and draw meaningful conclusions from the study.

## Author Contributions

**Houda Chtibi**: writing – original draft, data curation, formal analysis, visualization, validation, methodology, investigation, conceptualization. **Kaoutar Harboul**: methodology. **Taoufiq Benali**: conceptualization, validation, methodology, investigation, writing – review and editing, data curation. **Aziz Bouymajane**: methodology, investigation. **Roberto Laganà Vinci**: methodology, investigation. **Francesco Cacciola**: validation, methodology, writing – review and editing, supervision. **Luigi Mondello**: resources. **Khalil Hammani**: validation, supervision, writing – review and editing, resources, project administration, formal analysis, conceptualization.

## Conflicts of Interest

The authors declare no conflicts of interest.

## Data Availability

The data that support the findings of this study are available from the corresponding author upon reasonable request.
